# *Mychonastes homosphaera* MHSC24 Isolated from Brackish Waters of Korea: Taxonomic, Physiological, and Biochemical Characterization

**DOI:** 10.3390/microorganisms13102322

**Published:** 2025-10-07

**Authors:** Chang Rak Jo, Sangbum Lee, Ga Young Kim, Jeong-Mi Do, Ji Won Hong, Hae-Seo Noh, Hyung June Kim, Nam Seon Kang

**Affiliations:** 1 National Marine Biodiversity Institute of Korea, Seocheon 33662, Republic of Korea; happyccr@mabik.re.kr (C.R.J.); gykim@mabik.re.kr (G.Y.K.); kimhj95@mabik.re.kr (H.J.K.); 2 Center for Research Facilities, Kunsan National University, 558 Daehak-ro, Gunsan 54150, Republic of Korea; sblee08@kunsan.ac.kr; 3 Advanced Bio-Resource Research Center, Kyungpook National University, Daegu 41566, Republic of Korea; jmdoe09@knu.ac.kr (J.-M.D.); jwhong@knu.ac.kr (J.W.H.); noj224@naver.com (H.-S.N.); 4 Department of Biology, College of Natural Sciences, Kyungpook National University, Daegu 41566, Republic of Korea; 5 BK21 FOUR KNU Creative BioResearch Group, School of Life Sciences, Kyungpook National University, Daegu 41566, Republic of Korea

**Keywords:** *Mychonastes homosphaera*, Chlorophyta, canthaxanthin, protein-rich biomass, carotenoids, biotechnological potential

## Abstract

*Mychonastes homosphaera* MHSC24 is a green microalga newly isolated from a brackish coastal site in Korea. This study represents the first indigenous record of this species in the country. It provides a comprehensive characterization of its morphological, molecular, physiological, and biochemical characteristics. This microalga was identified through morphological observations and multilocus phylogenetic analyses. Strain MHSC24 exhibited robust growth under mesophilic temperatures (15–27 °C), moderate light intensities (88–300 μmol photons m^−2^ s^−1^), and low salinity levels (0–10 PSU). Optimal growth was observed at 27 °C, 193 μmol photons m^−2^ s^−1^, and 0 PSU. Under standard cultivation, the strain exhibited high protein levels (~54% of dry weight, DW) and accumulated substantial amounts of canthaxanthin (5.59 mg g^−1^ DW), the predominant carotenoid in its pigment profile. Thus, MHSC24 is a promising candidate for sustainable protein- and carotenoid-based applications. Palmitic acid (11.95 mg g^−1^ DW) and galactose (2.07 mg g^−1^ DW) were the predominant fatty acid and monosaccharide, respectively. The physiological resilience, high protein yield, and substantial canthaxanthin accumulation of MHSC24 support its potential utilization in the functional food, feed, and nutraceutical sectors. Therefore, this study provides a basis for optimized cultivation strategies and industrial exploitation of indigenous Korean microalgae.

## 1. Introduction

Increasing global challenges related to food security, climate change, and biodiversity conservation have accelerated the search for renewable and sustainable biological resources [1,2]. Microalgae have emerged as promising candidates because of their high photosynthetic efficiency, rapid biomass accumulation, and ability to produce numerous bioactive compounds of nutritional and industrial relevance [3,4,5].

Among microalgal metabolites, carotenoids have received particular attention as natural pigments with health-promoting properties. In particular, canthaxanthin is valued for its pigmentation role in aquaculture and animal feed, and for its expanding applications in the food and nutraceutical sectors [6,7]. In addition, protein-rich microalgae, such as *Chlorella* and *Tetradesmus*, have been commercialized as sustainable protein sources [8]. This demonstrates the importance of identifying new strains with high or superior protein content.

The Nagoya Protocol on Access and Benefit-Sharing emphasizes the importance of securing and characterizing novel microalgal strains by affirming national sovereignty over genetic resources and mandating their equitable utilization [9,10]. Consequently, the isolation and bioprospecting of indigenous strains have become strategic priorities for both biodiversity conservation and the development of sustainable bioindustries [11].

The genus *Mychonastes* (Chlorophyceae, Chlorophyta), established by Simpson and Van Valkenburg [12], comprises small coccoid, autosporic green algae that typically inhabit freshwater environments but are also occasionally found in brackish habitats. Species of this genus are morphologically characterized by spherical to ellipsoidal cells, parietal cup- or discoid-shaped chloroplasts lacking pyrenoids, and a strictly non-motile life cycle [12,13]. According to AlgaeBase (accessed 2025), the genus currently includes 27 accepted species [14].

Members of the *Mychonastes* genus are ecologically important components of freshwater phytoplankton that contribute to primary production and biodiversity [15,16]. They are also found in terrestrial and aerial habitats, which reflects their considerable ecological flexibility [17,18]. Their dominance in the picoplankton assemblages of certain lakes further illustrates their capacity to thrive under dynamic and nutrient-variable conditions [15].

Beyond their ecological roles, several *Mychonastes* species have demonstrated biotechnological potential. Their applications range from biofuel production and animal feed supplementation to carbon capture, pharmaceuticals, and water quality monitoring [19,20,21,22,23]. Among these species, *Mychonastes homosphaera* (Skuja) Kalina & Punčochářová [13] inhabits diverse freshwater [24,25] and terrestrial environments [18] across Europe, Asia, the Americas, Africa, Oceania, and even polar regions such as the Arctic and Antarctica [14,25,26,27,28,29,30]. Its broad distribution and adaptability make this species a promising resource for diverse industrial applications, including protein-rich biomass, high-value metabolites, and applications in wastewater treatment [20,31,32].

To our knowledge, there have been no reports of indigenous strains of *M. homosphaera* in Korea to date. However, microalgal strains within the same species display considerable variations in physiological traits and metabolite profiles [33,34]. Therefore, we aimed to characterize the native Korean strain *M. homosphaera* MHSC24, isolated from Janghang Harbor, Seocheon-gun. Specifically, the objectives of this study were to (1) confirm its taxonomic placement through morphological and molecular analyses; (2) evaluate its growth responses under different salinity, temperature, and light conditions; (3) analyze its biochemical composition, including pigment, fatty acid, and monosaccharide profiles.

As a previously unrecorded species in Korea, MHSC24 provides new insights into regional microalgal diversity. This study expands our understanding of Korean microalgal biodiversity and support the utilization of native *Mychonastes* strains in sustainable health-oriented biotechnology.

## 2. Materials and Methods

### 2.1. Sample Collection and Isolation

**Figure 1:**
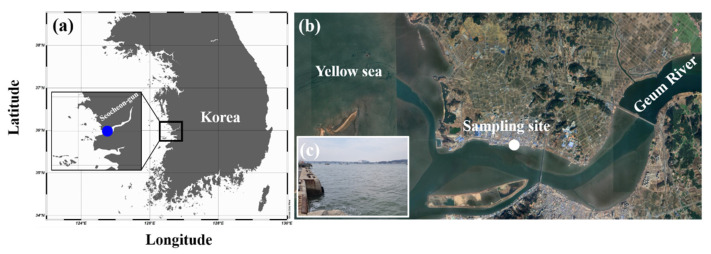
Sampling locations of *Mychonastes homosphaera* MHSC24 in Janghang Harbor, Seocheon-gun, Chungcheongnam-do, Korea. (**a**) A map showing the geographic location of Seocheon-gun on the west coast of Korea; (**b**) A satellite image of the sampling site at Janghang Harbor, influenced by freshwater inflow from the Geum River and tidal activity from the Yellow Sea (image obtained from Google Earth); (**c**) A photograph of the sampling site at Janghang Harbour, where *M. homosphaera* MHSC24 was collected.

In April 2024, plankton samples were collected from the brackish estuary of Janghang Harbor (36°00′24.69″ N, 126°41′40.03″ E) in Seocheon-gun, Chungcheongnam-do, Korea. At the time of collection, the water temperature and practical salinity unit (PSU) were 11.6 °C and 7.5, respectively (Table 1, Figure 1). Unialgal cultures were established as described by Kang et al. [35]. The isolated algal strain was maintained on BG-11 agar plates at 27 °C in a plant growth chamber (JSR, Gongju, Korea) under a 14 h light:10 h dark cycle; cool-white fluorescent lights provided an irradiance of 170 μmol photons m^−2^ s^−1^.

### 2.2. Morphological Identification

The morphology of living cells grown photosynthetically was examined using an inverted microscope (CKX53; Olympus, Tokyo, Japan). The length and width of live cells were measured using a digital camera (AxioCam MRc5; Carl Zeiss, Göttingen, Germany). To analyze colony morphology, exponentially growing cultures were diluted 10-fold with fresh BG-11 medium, and 0.1 mL of the suspension was spread evenly onto 10 mL of BG-11 medium solidified with 1.0% agar in 90 × 10 mm Petri dishes. The plates were incubated at 27 °C under a 14:10 h light:dark photoperiod; cool-white fluorescent lights were used to provide an irradiance of 170 μmol photons m^−2^ s^−1^. After 21 days, the colony morphology was examined using the same inverted microscope equipped with 4× or 10× objective lenses. Field emission scanning electron microscopy and transmission electron microscopy (TEM) were performed as described by Kang et al. [35].

### 2.3. Molecular Identification and Sequence Analysis

Genomic DNA was extracted, amplified using PCR, and sequenced as described by Kang et al. [35] and Jo et al. [36]. The nucleotide sequences were compared with those in the National Center for Biotechnology Information GenBank database using the Basic Local Alignment Search Tool. Sequence alignment and phylogenetic analyses were performed using Geneious Prime v2024.0.7 (Biomatters Ltd., Auckland, New Zealand), with reference sequences obtained from GenBank (18S rDNA: 16 reference sequences with Hydrodictyaceae sp. HND10-6 [MH176112] as the outgroup; ITS1–5.8S–ITS2: 23 reference sequences with *Chloromonas rosae* CCAP 11/112 [FR865528] as the outgroup; *rbc*L: 17 reference sequences with *Characiochloris sasae* NIES-567 [AB084338] as the outgroup).

Bayesian phylogenetic trees were reconstructed using MrBayes v.3.2.7 [37,38]; four Markov chain Monte Carlo chains were run for each dataset, as described by Kang et al. [39]. In addition, maximum likelihood (ML) analyses were conducted using raxmlGUI 2.0 [40]. Both Bayesian and ML analyses were performed under the GTR + G + I substitution model. Two hundred independent inferences were made using the –# option to identify the optimal tree, and bootstrap values were calculated with 1000 replicates.

### 2.4. Determination of Optimal Culture Conditions

To determine the optimal salinity for *M. homosphaera* MHSC24, the BG-11 medium was prepared by diluting sterilized natural seawater with deionized water to achieve final salinity levels of 0, 5, 10, 15, 20, 25, and 30 PSU. All media were sterilized by filtration and autoclaving before use. Cultures were inoculated at an initial cell density of 1 × 10^6^ cells mL^−1^ and incubated in an incubator (EYELA, Bunkyo-ku, Tokyo, Japan) at 27 °C, with continuous shaking at 150 rpm for acclimation over 21 days under a 14:10 h light:dark photoperiod. Cool-white fluorescent lights provided an irradiance of approximately 170 μmol photons m^−2^ s^−1^.

Following acclimation, growth optimization was performed under different salinity conditions. Cultures acclimated well and reached sufficient cell densities under low-salinity conditions (0–15 PSU). The acclimated cultures were then used for further experiments. In contrast, growth was barely achieved at higher salinities (20–30 PSU). Consequently, stable acclimation was not possible. Therefore, experiments at these salinity levels were conducted using cultures pre-acclimated at 15 PSU. All cultures were re-inoculated at an initial cell density of 1 × 10^6^ cells mL^−1^ and incubated at 27 °C under the same light regime and irradiance, with continuous shaking at 150 rpm.

Growth was monitored daily for 24 days by measuring the optical density at 600 nm (OD_600_) using a Synergy II microplate reader (BioTek, Winooski, VT, USA). Cell density was estimated using a pre-established calibration curve relating OD_600_ values to actual cell concentrations. Based on these results, 0 PSU was identified as the optimal salinity and was used in all subsequent experiments.

Temperature and light intensity were optimized using cultures maintained at 0 PSU in a PhotoBiobox system (London, UK) [41]. A 200 μL aliquot of culture, adjusted to an initial cell density of 1 × 10^6^ cells mL^−1^, was dispensed into 96-well black/clear-bottom microplates, sealed with adhesive plate film, and incubated for 72 h across a range of temperatures (5–40 °C) and light intensities (0–350 μmol photons m^−2^ s^−1^). After incubation, the OD_600_ was measured using the same microplate reader. OD_600_ values were used as a proxy for biomass, and the specific growth rate (μ) was calculated using the following equation:

μ = (ln *A*_2_ − ln *A*_1_)/(*T*_2_ − *T*_1_)

where *A*_1_ and *A*_2_ represent the OD_600_ values at 0 and 72 h, respectively. The calculated growth rates were visualized as heat maps using Microsoft Excel 2019 (Microsoft Corp., Redmond, WA, USA).

### 2.5. Determination of Total Lipid, Carbohydrate, and Protein Contents

Cultures were grown under standard conditions (0 PSU BG-11 medium, 1 × 10^6^ cells mL^−1^, 27 °C, continuous shaking at 150 rpm, 14 h light: 10 h dark photoperiod, 170 μmol photons m^−2^ s^−1^) for 21 days. Biomass harvested in the late exponential phase was freeze-dried using a FreeZone 4.5 freeze dryer (Labconco, Kansas City, MO, USA) for biochemical analysis.

The total carbohydrate content was determined by resuspending 5–6 mg of lyophilized algal biomass in 25 mL of distilled water. A 1 mL aliquot of the suspension was mixed with 1 mL of 5% phenol and 5 mL of concentrated sulfuric acid, following the phenol–sulfuric acid method described by DuBois et al. [42]. After cooling in a cold-water bath, absorbance was measured at 488 nm using a Synergy II microplate reader. The carbohydrate concentration was calculated using a standard glucose curve.

Total protein content was estimated from the nitrogen content using elemental analysis at 950 °C with a Leco FP-528 N determinator (LECO Corporation, St. Joseph, MI, USA). Ultra-high-purity oxygen and helium were used as the combustion and carrier gases, respectively. The nitrogen-to-protein conversion factor was determined as described by Mariotti et al. [43].

The total lipid content was measured from the dried biomass using a methanol–chloroform extraction method, as described by Kim et al. [44].

### 2.6. Microalgal Pigment Extraction and Analysis

Freeze-dried biomass obtained under the standard cultivation conditions described in Section 2.5 was used for pigment extraction. Approximately 10 mg of freeze-dried biomass was accurately weighed and resuspended in methanol (1.5 mL).

The samples were sonicated in an ultrasonic bath (Bransonic CPX5800H-E; Branson, Danbury, CT, USA) at 40 Hz for 90 min and subsequently centrifuged at 16,000× *g* for 20 min at 4 °C. The resulting supernatant was collected and dried using a rotary evaporator (IKA RV; IKA, Staufen, Germany). The residue was redissolved in 1.5 mL of acetone and filtered through a 0.2 μm membrane filter (Minisart syringe filter; Sartorius, Göttingen, Germany) before high-performance liquid chromatography (HPLC) analysis.

Carotenoid analysis was performed using an Agilent 1200 series HPLC system (Agilent Technologies, Palo Alto, CA, USA) equipped with a C30 carotenoid column (250 mm × 4.6 mm, 5 μm; YMC, Kyoto, Japan), as described by Yang et al. [45]. The mobile phase consisted of solvent A (92% methanol containing 10 mM NH_4_CH_3_CO_2_) and solvent B (tert-butyl methyl ether), delivered at a flow rate of 1 mL min^−1^ for 60 min under gradient conditions.

Carotenoid standards, including astaxanthin, β-carotene, canthaxanthin, lutein, and zeaxanthin (Sigma-Aldrich, St. Louis, MO, USA), were used to identify and quantify pigment profiles. Detection was performed at 450 nm by comparing the retention times and absorption spectra of standards and samples.

### 2.7. Analyses for Fatty Acid Composition of Lipids

The freeze-dried biomass obtained under standard conditions (Section 2.5) was used to analyze fatty acid composition. Total lipids were extracted from the freeze-dried biomass and converted to fatty acid methyl esters (FAMEs) using standard transesterification procedures. The resulting FAMEs were analyzed using a 7890A gas chromatograph coupled to a 5975C mass selective detector (Agilent Technologies, Santa Clara, CA, USA), as described by Oliveira et al. [46].

The fatty acid compounds were identified by comparing the obtained mass spectra with the reference spectra in the Wiley/NBS mass spectral library. Only peaks with a similarity index >90% were used for compound identification.

### 2.8. Microalgal Monosaccharide Extraction and Analysis

The freeze-dried biomass of *M. homosphaera* MHSC24 obtained under standard conditions (Section 2.5) was used to analyze the monosaccharide composition. Freeze-dried biomass (50 mg) was hydrolyzed in 2.5 mL of 2 N sulfuric acid at 94 °C for 3 h. After cooling to room temperature, the hydrolysate was neutralized with calcium carbonate (CaCO_3_) and filtered through a 0.2 μm PTFE membrane filter (Whatman, Maidstone, UK).

The monosaccharide composition was analyzed using an Alliance HPLC system (Waters Co., Milford, MA, USA) equipped with a Sugar-Pak I column (6.5 × 300 mm; Waters Co.) and a refractive index detector(Waters Co., Milford, MA, USA). The mobile phase consisted of 0.01 M calcium-EDTA solution (50 mg L^−1^ in distilled water), with a flow rate of 0.5 mL min^−1^ and a column temperature of 90 °C. A 20 μL aliquot of each sample was injected.

Individual monosaccharides were identified and quantified through comparison with authentic standards (sucrose, lactose, glucose, galactose, fructose, arabinose, mannitol, and sorbitol; Sigma-Aldrich). Monosaccharide content is expressed as mg g^−1^ dry weight (DW).

### 2.9. Statistical Analysis

All experiments were conducted in triplicate. The data are presented as the mean ± standard deviation (SD) and, where indicated, the standard error (SE). Statistical analyses were performed using one-way analysis of variance (ANOVA), followed by Tukey’s honest significant difference (HSD) post hoc test to evaluate differences among the means. Before ANOVA and Tukey’s HSD test, the normality of the data was assessed using the Shapiro–Wilk test. Statistical significance was set at *p* < 0.05. All analyses were performed using SPSS Statistics v14.0 (SPSS Inc., Chicago, IL, USA).

## 3. Results

### 3.1. Morphological Characteristics

**Figure 2:**
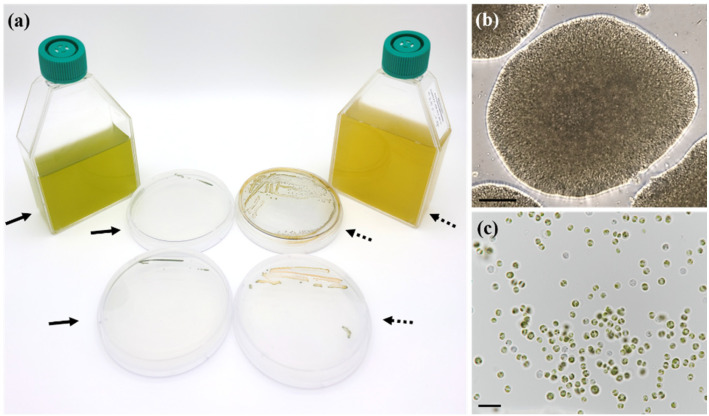
Growth-associated color changes and cellular morphology of *M. homosphaera* MHSC24. (**a**) Progressive color changes in cultures. Initially, both the liquid and solid BG-11 media appeared green (arrows), which is a characteristic of active cell growth. The cultures turned yellowish-brown (dashed arrows) after 2–3 months of incubation, indicating the onset of the stationary phase or senescence; (**b**) A densely packed colony formed on a solid BG-11 agar plate; (**c**) Light micrographs showing *M. homosphaera* MHSC24 cells exhibiting a range of sizes and life stages. Scale bars: (**b**) = 100 μm, (**c**) = 10 μm.

*M. homosphaera* MHSC24 cells were typically green during active growth. However, they gradually turned yellowish-brown as the cultures aged (Figure 2a). A color change was observed in both liquid and solid BG-11 media, indicating a transition from exponential to stationary phases. On the BG-11 agar plates, the strain formed compact circular colonies with smooth or slightly undulating margins (Figure 2b). Light microscopy revealed densely packed cells within the colonies, particularly along the edges.

**Figure 3:**
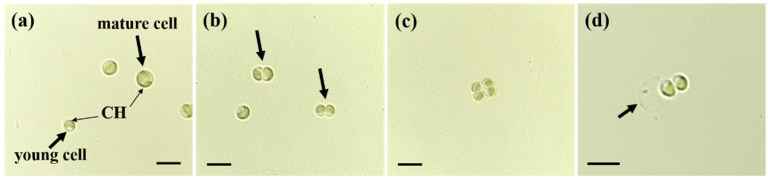
Light micrographs of *M. homosphaera* MHSC24. (**a**) Young and mature vegetative cells, both exhibiting a single chloroplast (CH). The young cell is smaller and less developed than the mature cell; (**b**) An autosporangium containing two autospores, indicated by arrows; (**c**) An autosporangium containing four autospores; (**d**) Release of autospores from the mother cell. Two autospores can be seen outside, and the remnant mother cell wall is indicated by an arrow. Scale bars: (**a**–**d**) = 5 μm.

Both young and mature cells were predominantly solitary in liquid culture; frequent autosporangia and considerable variations in cell size and morphology were observed (Figure 2c and Figure 3). Young cells were nearly spherical or ovoid. Spherical cells measured 1.43–2.67 μm in diameter (mean ± SE: 2.10 ± 0.08 μm; n = 30) (Table 2). Ovoid young cells measured 1.25–2.75 μm in length (1.93 ± 0.08 μm) and 1.00–2.38 μm in width (1.61 ± 0.07 μm).

Mature cells were spherical or ovoid, but larger than young cells. Spherical cells measured 2.78–4.02 μm in diameter (mean ± SE: 3.46 ± 0.07 μm), whereas ovoid cells measured 2.63–3.88 μm in length (3.04 ± 0.07 μm) and 2.38–3.63 μm in width (2.76 ± 0.07 μm) (Table 2).

Asexual reproduction occurred via autosporulation. Successive bipartitions of the protoplast generally resulted in two or four autospores within the mother cell (Figure 3b,c), with up to six rarely observed. Apertures through which the autospores were discharged were frequently observed in the mother cell wall (Figure 3d). Most cells, regardless of the developmental stage, contained a single parietal, cup-shaped chloroplast lacking a pyrenoid (Figure 3).

**Figure 4:**
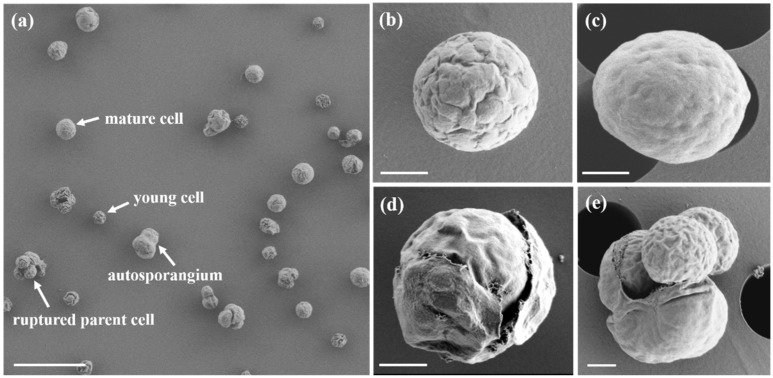
Scanning electron micrographs of *M. homosphaera* MHSC24. (**a**) An overview of cells of different sizes and life stages, including young and mature cells, autosporangia, and ruptured parental cells; (**b**) A young spherical cell with a sculptured surface showing pronounced undulations; (**c**) A mature cell with a relatively smooth surface and visible residual undulations; (**d**) An autosporangium with a ruptured cell wall containing autospores; (**e**) Two autospores are visible outside the ruptured mother cell wall after release. Scale bars: (**a**) = 10 μm, (**b**–**d**) = 1 μm.

SEM revealed the surface features of young and mature cells, as well as those of the autosporangia (Figure 4). Both young and mature cells were nearly spherical or ovoid (Figure 4a–c), with surface textures ranging from distinctly undulated in young cells to relatively smooth in mature cells (Figure 4b,c). Some cells displayed ruptured mother cell walls with autospores retained inside (Figure 4d), whereas others showed daughter cells that were already released (Figure 4e).

**Figure 5:**
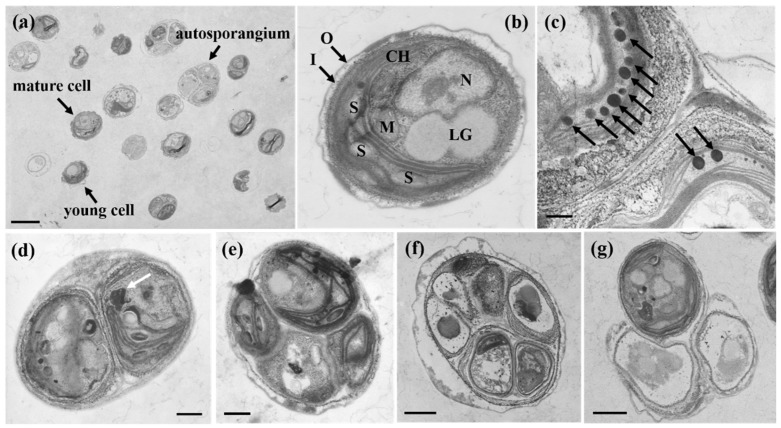
Transmission electron micrographs of *M. homosphaera* MHSC24. (**a**) An overview of cells at different life cycle stages, including young cells, mature cells, and autosporangia; (**b**) A single cell showing internal organelles, including a cup-shaped chloroplast (CH), lipid globules (LG), the nucleus (N), the mitochondrion (M), and multiple starch granules (S). The arrow indicates a double-layered cell wall with inner (I) and outer (O) layers; (**c**) Electron-dense plastoglobules (arrows) located adjacent to the thylakoid membranes are commonly observed during the stationary phase; (**d**) Autosporangium with two developing autospores. The arrow indicates an electron-dense body; (**e**) Autosporangium with four developing autospores; (**f**) Autosporangium with six developing autospores; (**g**) Release of a single autospore from a ruptured mother cell wall. Scale bars: (**a**) = 3 μm, (**f**,**g**) = 1 μm, (**d**,**e**) = 0.5 μm, (**b**,**c**) = 0.3 μm.

TEM revealed the ultrastructural features of cells at different developmental stages (Figure 5). The cells were spherical or ovoid (Figure 5a). The parietal cup-shaped chloroplast (CH) lined the inner cell wall, and the nucleus (N) was located laterally (Figure 5b). Mitochondria (M) and starch granules (S) were observed; starch was distributed among the thylakoid lamellae (Figure 5b). Pyrenoids were absent from the chloroplasts. The cell wall consisted of the outer (O) and inner (I) layers (Figure 5b, arrows), whereas the cytoplasm contained lipid globules (LG). Electron-dense plastoglobules were observed within the chloroplast and occasionally between the thylakoid membranes (Figure 5c). Electron-dense cytoplasmic inclusions were also observed (Figure 5d, arrow). Autosporulation was evident, with 2–6 autospores developing inside the mother cell (Figure 5d–f). Cells in which autospores were released through rupture of the maternal cell wall were also observed (Figure 5g).

### 3.2. Molecular Identification and Sequence Analysis

The combined length of the small-subunit (SSU) rDNA, internal transcribed spacer (ITS1–5.8S–ITS2), large subunit (LSU; 28S rDNA) region, *rbc*L (encoding the large subunit of ribulose-1,5-bisphosphate carboxylase/oxygenase), and *tuf*A (encoding elongation factor Tu) sequences of the newly isolated strain MHSC24 was 4434 bp (GenBank accession numbers: PV432769, PV432770, PV432771, PV430742, and PV430743; Table 1).

Sequence alignment revealed that the SSU rDNA sequence of *M. homosphaera* MHSC24 was identical to that of strains CCALA 380, CAUP H6501, and CCAP 205/1; only a 0–1 bp difference was observed (Table 3). Similarly, the ITS1–5.8S–ITS2 region of MHSC24 was identical to that of strain NIES-4546 but differed from those of other strains by 2–22 bp. The LSU, *rbc*L, and *tuf*A sequences also showed complete identity or minor variations (1–5 bp) compared with the reference strains (Table 3).

**Figure 6:**
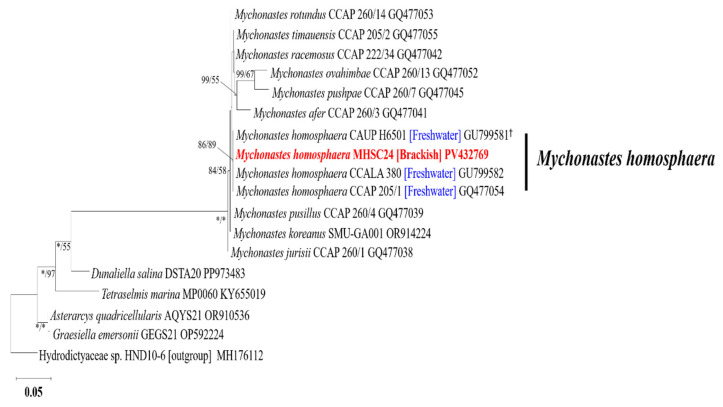
Maximum likelihood (ML) and Bayesian inference (BI) phylogenetic trees were constructed using 18S rDNA sequences. The numbers at each node represent the ML bootstrap values (left) and BI posterior probabilities (right). Bootstrap values <50% are not shown. Posterior probabilities are shown for the major nodes, with an asterisk (*) indicating full support (1.00). The strain analyzed in this study (*M. homosphaera* MHSC24) is highlighted in red. Blue indicates freshwater strains. The dagger symbol (†) denotes the authentic strain. The scale bar represents the number of nucleotide substitutions per site.

MHSC24 clustered with other *M. homosphaera* strains in the SSU rDNA-based phylogenetic tree, including the authentic strain CAUP H6501 and the reference strains CCALA 380 and CCAP 205/1 (Figure 6). In the ITS1–5.8S–ITS2 rDNA-based tree, MHSC24 clustered within a well-supported clade comprising NIES-4546, CCAP 205/1, NIES-2341, CCALA 380, and CAUP H6501 (Figure S1). Similarly, MHSC24 clustered with GA11, GA13, CAUP H6502, NIES-4546, and NIES-2341 in the *rbc*L-based tree (Figure S2).

Molecular phylogenetic analyses using SSU rDNA, ITS1–5.8S–ITS2, and *rbc*L sequences confirmed that this isolate belonged to *M. homosphaera*. Thus, the strain was designated *M. homosphaera* MHSC24 and has been deposited in the National Marine Biodiversity Institute of Korea (MABIK) and the Korean Collection for Type Cultures (KCTC) under accession numbers MABIK FL00031124 and KCTC 16415BP, respectively.

### 3.3. Verification of the Optimal Cultivation Conditions of the Isolated Strain

**Figure 7:**
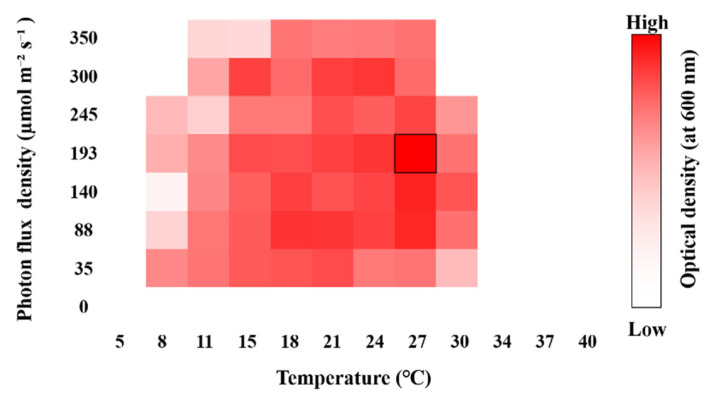
A heatmap showing the growth response of *M. homosphaera* MHSC24 under different temperature and light intensity conditions. Cells were cultured using the PhotoBiobox system. Color intensity corresponds to optical density (OD_600_), ranging from low (white) to high (red). The black box indicates the condition with the highest OD_600_ value (27 °C, 193 μmol m^−2^ s^−1^).

Growth responses of the isolated algal strains were assessed under laboratory conditions to determine the optimal cultivation parameters, namely, salinity, temperature, and light intensity. As shown in Figure 7, *M. homosphaera* MHSC24 exhibited high growth rates at 15–27 °C and under photon flux densities (PFDs) of 88–300 μmol photons m^−2^ s^−1^. The optimal growth conditions were determined to be 27 °C and 193 μmol photons m^−2^ s^−1^.

**Figure 8:**
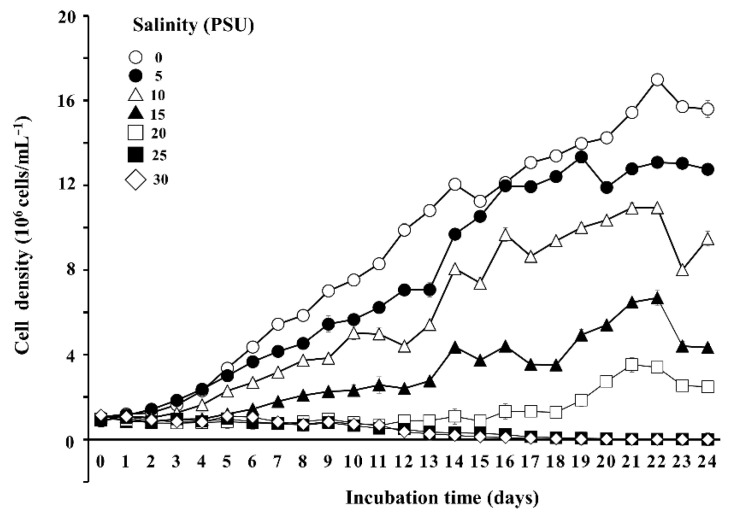
The growth response of *M. homosphaera* MHSC24 under different salinities (0–30 PSU) during a 24-day cultivation period. Data are presented as the mean ± standard deviation (SD) (*n* = 3).

As shown in Figure 8, seawater salinity had a marked effect on the density of *M. homosphaera* MHSC24 cells during the 24-day cultivation period. The strain exhibited active, moderate, and limited growth at 0–10, 15, and 20 PSU, respectively. Contrastingly, no growth was detected at 25 and 30 PSU. At 0 PSU, cultures reached a maximum cell density of approximately 17 × 10^6^ cells mL^−1^ by day 22.

### 3.4. Proximate Composition of Dried M. homosphaera Biomass

**Figure 9:**
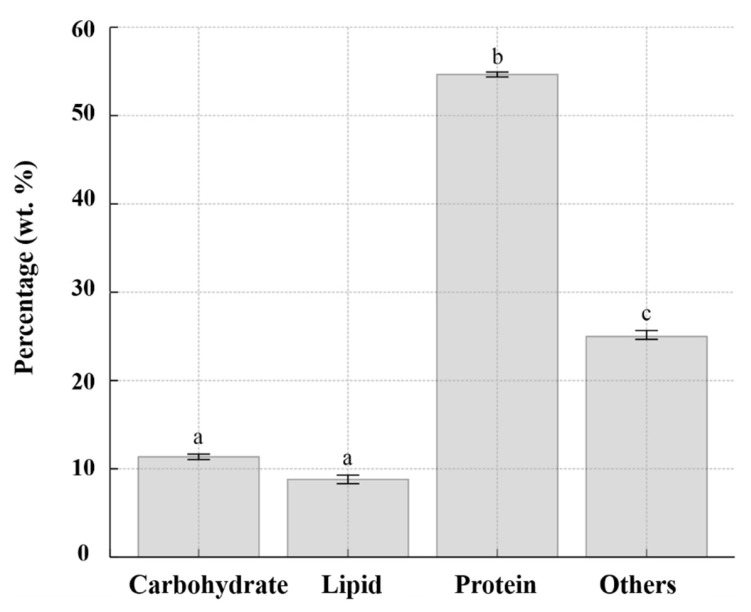
Proximate composition of *M. homosphaera* MHSC24 cultivated in BG-11 medium under optimal conditions. Bars represent the mean values of total carbohydrates, lipids, proteins, and other components, expressed as weight percentages (wt%). Error bars indicate the mean ± SD (n = 3). Different lowercase letters above the bars denote statistically significant differences among the groups, as determined using Tukey’s honest significant difference test (*p* < 0.05).

The proximate composition of *M. homosphaera* MHSC24, based on biomass cultivated under standard conditions (0 PSU BG-11, 10^6^ cells mL^−1^, 27 °C, 14:10 h photoperiod, 170 μmol photons m^−2^ s^−1^, 150 rpm, for 21 days), is shown in Figure 9. Proteins constituted the predominant fraction, accounting for approximately 54% of the dry weight, whereas carbohydrates and lipids accounted for approximately 12% and 9% of the dry weight, respectively. 

### 3.5. Analysis of Microalgal Pigment Profile

The pigment profile of *M. homosphaera* MHSC24, as determined based on the biomass cultivated under the standard conditions described in Section 3.4, is summarized in Table 4. Twelve pigments, including chlorophyll, xanthophyll, and carotene, were identified. Among the carotenoids, canthaxanthin had the highest content (5.59 mg g^−1^), followed by lutein (3.64 mg g^−1^). Chlorophyll *a* exhibited the highest concentration among all pigments (11.42 mg g^−1^), whereas chlorophyll *b* was also present at a moderate level (2.50 mg g^−1^). Minor pigments, including violaxanthin (0.61 mg g^−1^), echinenone (0.50 mg g^−1^), β-carotene (0.49 mg g^−1^), neoxanthin (0.48 mg g^−1^), and antheraxanthin (0.13 mg g^−1^), were also detected.

### 3.6. Fatty Acid Composition of Lipids

The fatty acid composition of *M. homosphaera* MHSC24, as determined based on biomass cultivated under the standard conditions described in Section 3.4, is presented in Table 5. FAME analysis revealed that the predominant fatty acids were palmitic acid (C_16:0_; 39.72%, 11.95 mg g^−1^ DW), oleic acid (C_18:1_ n-9; 17.28%, 5.20 mg g^−1^ DW), α-linolenic acid (C_18:3_ n-3; 15.61%, 4.70 mg g^−1^ DW), and lignoceric acid (C_24:0_; 11.07%, 3.33 mg g^−1^ DW). Nervonic acid (C_24:1_; 6.98%, 2.10 mg g^−1^ DW) and linoleic acid (C_18:2_ n-6; 6.28%, 1.89 mg g^−1^ DW) were also detected at moderate levels. Minor fatty acids included stearic acid (C_18:0_; 2.17%, 0.65 mg g^−1^ DW) and eicosapentaenoic acid (C_20:5_ n-3; 0.88%, 0.27 mg g^−1^ DW). Furthermore, saturated fatty acids constituted 52.96% of the total FAMEs (15.93 mg g^−1^ DW), followed by monounsaturated fatty acids at 24.26% (7.30 mg g^−1^ DW) and polyunsaturated fatty acids at 22.77% (6.85 mg g^−1^ DW).

### 3.7. Analysis of the Monosaccharide Profile

The monosaccharide composition of *M. homosphaera* MHSC24, as determined based on the biomass cultivated under the standard conditions described in Section 3.4, is shown in Table 6. Galactose was the predominant monosaccharide (2.07 mg g^−1^ DW). Sucrose (0.99 mg g^−1^ DW) and glucose (0.75 mg g^−1^ DW) were also detected, followed by fructose (0.62 mg g^−1^ DW) and mannitol (0.46 mg g^−1^ DW).

## 4. Discussion

This study reports the characteristics of *M. homosphaera* MHSC24, a newly recorded species in Korea. This strain was validated using integrated morphological and molecular analyses. It exhibited broad tolerance to salinity, temperature, and irradiance, reflecting ecological adaptability. Moreover, *M. homosphaera* MHSC24 was characterized by high protein content and a distinctive pigment profile with substantial canthaxanthin accumulation. These characteristics highlight MHSC24 as a taxonomically validated and biochemically valuable microalga with a strong potential for food and feed applications.

### 4.1. Morphological and Ultrastructural Traits with Pigment Accumulation

The morphological characteristics of MHSC24 were largely consistent with the original description of the genus *Mychonastes* by Simpson and Van Valkenburg [12]. The strain exhibited green autosporic cells with spherical to ovoid shapes, parietal cup-shaped chloroplasts lacking pyrenoids, and double-layered cell walls with irregular surface ornamentation. Asexual reproduction occurred via autosporulation, typically producing two–four autospores per mother cell. It occasionally produced up to six autospores, consistent with the genus-level feature of forming 2–64 autospores per cell. Most cells were solitary or formed small aggregates, and no motile stages were observed. Ultrastructural observations revealed starch granules within chloroplasts and laterally positioned nuclei. These features closely resembled those of other *M. homosphaera* strains such as CCAP 211/8e from Sweden and the ND strain from Israel [24]. Additionally, the observed cell dimensions of MHSC24 were within the intraspecific variation reported for this species (Table 2). MHSC24 cells formed compact circular colonies with smooth or slightly undulating margins on BG-11 solid medium (Figure 2b). These characteristics were consistent with the Type 1 colony pattern of *M. homosphaera* described by Nozaki et al. [25].

As MHSC24 cultures entered the stationary phase, a marked shift in coloration from green to yellowish-brown was observed in both the liquid and solid BG-11 cultures (Figure 2a). These pigmentation changes are commonly observed in green microalgae during the late cultivation stages [35,49,50]. For instance, aging cultures accumulate secondary carotenoids in *Mychonastes frigidus*, resulting in yellow, orange, or brown coloration [51]. This phenomenon is widespread in Chlorophyta and redirects metabolic flux toward carotenoid biosynthesis under prolonged culture or nutrient-depleted conditions [52,53,54].

This pigmentation shift likely reflects underlying stress responses, as supported by ultrastructural observations. Electron-dense plastoglobules were frequently observed within chloroplasts during the late growth phase; these were particularly adjacent to the thylakoid membranes (Figure 5c). Plastoglobules are lipid-rich suborganellar compartments that typically proliferate under stress conditions, such as high light exposure in *Dunaliella bardawil* [55] or dehydration in *Tetradesmus* spp. [56]. Furthermore, they act as dynamic reservoirs for carotenoids, tocopherols, and other lipophilic antioxidants. Although no intentional stress treatments were applied in this study, the accumulation of plastoglobules in MHSC24 cells during prolonged cultivation suggests chloroplast remodeling and intracellular sequestration of carotenoids or other antioxidant metabolites. These observations indicate that MHSC24 cells can adapt to progressive metabolic stress or nutrient limitation during extended growth.

### 4.2. Molecular Identification and Phylogenetic Analysis

Multilocus sequence analysis was performed using nuclear ribosomal regions (SSU, ITS1–5.8S–ITS2, and LSU) and chloroplast-encoded genes (*rbc*L and *tuf*A) to confirm the species-level identity of MHSC24. Sequence alignments revealed near-complete identity with previously characterized *M. homosphaera* strains, showing only 0–1 bp differences across most loci (Table 3). The ITS1–5.8S–ITS2 region was identical to that of the freshwater strain NIES-4546, whereas other reference strains differed by up to 22 bp, thereby confirming the conspecificity of MHSC24.

Phylogenetic reconstructions based on both ML and Bayesian inference consistently placed MHSC24 within the well-supported *M. homosphaera* clade across all analyzed markers (Figure 6; Figures S1 and S2). In the SSU- and ITS-based trees, MHSC24 clustered with high support alongside the authentic strain CAUP H6501 and freshwater-origin strains CCALA 380 and CCAP 205/1. The *rbc*L-based phylogeny similarly reinforced this classification, confirming the close evolutionary relationship between MHSC24 and the established freshwater representatives of the species.

Although most known strains of *M. homosphaera* have been isolated from freshwater environments, MHSC24 was collected from a brackish coastal site in Korea. Nevertheless, this isolate was phylogenetically grouped with freshwater strains, suggesting that salinity tolerance may occur in certain *M. homosphaera* ecotypes. This ecological plasticity is consistent with previous reports on *Mychonastes* species inhabiting diverse habitats, including soils, terrestrial surfaces, oligotrophic lakes, and high-irradiance aquatic systems [17,18,25,57]. The type species *M. ruminatus* was originally described in brackish water [12], indicating that halotolerance is not unprecedented within the genus. The ability of MHSC24 to grow efficiently at low salinity (0 PSU) further emphasizes its physiological versatility and ecological relevance in transitional aquatic systems.

Taken together, the morphological, ultrastructural, and multilocus phylogenetic evidence robustly supports the identification of MHSC24 as *M. homosphaera* and expands the known biogeographical and ecological range of this species to include brackish coastal environments in Korea.

### 4.3. Environmental Tolerance and Cultivation Potential

Growth responses were assessed at varying temperatures, PFDs, and salinity levels to evaluate the physiological characteristics of *M. homosphaera* MHSC24 (Figure 7 and Figure 8). The strain exhibited high growth rates at 15–27 °C and at PFDs of 88–300 μmol photons m^−2^ s^−1^. Optimal conditions were determined to be 27 °C and 193 μmol photons m^−2^ s^−1^ (Figure 7). These results indicate that MHSC24 is well-suited for mesophilic temperature and moderate light intensity conditions, showing adaptability across a practical cultivation range. The strain also retained measurable growth outside the optimal range, supporting its applicability under the environmental variability commonly encountered in semi-controlled or outdoor systems.

An *M. homosphaera* strain from Lake Kinneret, Israel, exhibited optimal growth at 14–20 °C, with a marked decline at temperatures >28 °C [58]. Similarly, MHSC24 displayed robust growth at 15–27 °C but also sustained detectable proliferation at 30 °C. This indicates enhanced tolerance to relatively higher temperatures. Although the Israeli strain showed photoinhibition under high irradiance (>700 μmol photons m^−2^ s^−1^), MHSC24 maintained consistent growth across moderate-to-high PFDs (88–300 μmol photons m^−2^ s^−1^) within the tested range; no clear signs of photoinhibition were observed. These findings suggest intraspecific variation in both thermal and photophysiological tolerance within the *M. homosphaera* clade and highlight the suitability of MHSC24 for moderately warm and illuminated cultivation conditions.

In terms of the salinity response, MHSC24 exhibited traits typical of freshwater ecotypes. Despite being isolated from a brackish coastal environment, the strain achieved its highest cell density at 0 PSU (~17 × 10^6^ cells mL^−1^ on day 22) and maintained moderate growth at 5–10 PSU (Figure 8). Its growth was strongly inhibited at 15 PSU, with almost no proliferation observed at 25–30 PSU. This restricted halotolerance was consistent with its phylogenetic placement within the primarily freshwater lineage of *M. homosphaera*.

Overall, MHSC24 demonstrated tolerance to key abiotic factors, including temperature, PFD, and salinity, within the ranges relevant for practical cultivation. Its reliable growth under mesophilic and moderate PFD conditions, combined with modest halotolerance, highlights its suitability for outdoor or semi-controlled mass cultivation systems subject to seasonal and climatic fluctuations. This physiological robustness both reinforces the potential of MHSC24 as a candidate for large-scale freshwater-based applications and highlights its ecological versatility and biotechnological utility.

### 4.4. Biochemical Profile and Functional Applications

Proximate biochemical analysis of *M. homosphaera* MHSC24 revealed a macronutrient profile characterized by a high protein content. This accounted for ~54% of the dry biomass, followed by carbohydrates (~12%) and lipids (~9%) (Figure 9). This value is markedly higher than the average protein content of most green microalgae, which typically accounts for approximately 30% of the dry weight [59].

MHSC24 demonstrated a markedly superior protein profile compared to those of previously reported strains within the genus *Mychonastes*, such as *M. homosphaera* QUCCCM70 (39.1%), LEAF0708 (12.6%), and *M. afer* PKUAC 9 (~20.9%) [20,60,61].

MHSC24 also performed favorably compared to commercially established taxa. *Chlorella vulgaris* (51–58%) and *Tetradesmus obliquus* [=*Scenedesmus obliquus*] (50–56%) are protein-rich microalgae that are widely cultivated for food and feed [8]. Although the MHSC24 protein content was slightly below the upper range of these species, it achieved this level under standard, non-stress conditions. This is contrary to some taxa that require nutrient deprivation or high light to accumulate elevated protein levels [8]. This stability under moderately scalable regimes highlights the biochemical efficiency and production reliability of MHSC24.

The protein content of this strain surpasses that of conventional dietary protein sources such as rice, milk, soy, meat, and eggs [62]. In addition, microalgae offer distinct advantages for sustainable biomass production, including rapid growth, year-round cultivation, use of non-arable land, and compatibility with saline water or wastewater [63,64].

In addition to bulk nutrition, microalgal proteins also exhibit functional properties. Enzymatic hydrolysates of microalgal proteins generate antioxidant peptides with radical scavenging activity [65,66]. Moreover, they are enriched in essential and branched-chain amino acids that support immune function and muscle metabolism [67]. However, further investigation of the peptide profile of MHSC24 is required to evaluate its nutraceutical potential.

Taken together, these findings suggest that *M. homosphaera* MHSC24 is a promising candidate for functional food, nutraceutical, and aquafeed applications. Its high and stable protein content, competitiveness with leading microalgal taxa, and suitability for scalable freshwater cultivation collectively highlight its value for sustainable high-protein biomass production.

### 4.5. Pigment Profile and Canthaxanthin Biosynthetic Potential of MHSC24

The pigment analysis of *M. homosphaera* MHSC24 cultivated under optimal salinity conditions (0 PSU) revealed diverse carotenoid profiles. The most prominent compounds were canthaxanthin (5.59 mg g^−1^ DW) and lutein (3.64 mg g^−1^ DW), along with lower concentrations of zeaxanthin, violaxanthin, β-carotene, and other minor pigments (Table 4). Canthaxanthin was the predominant carotenoid, accounting for a substantial proportion of the pigment pool.

**Figure 10:**
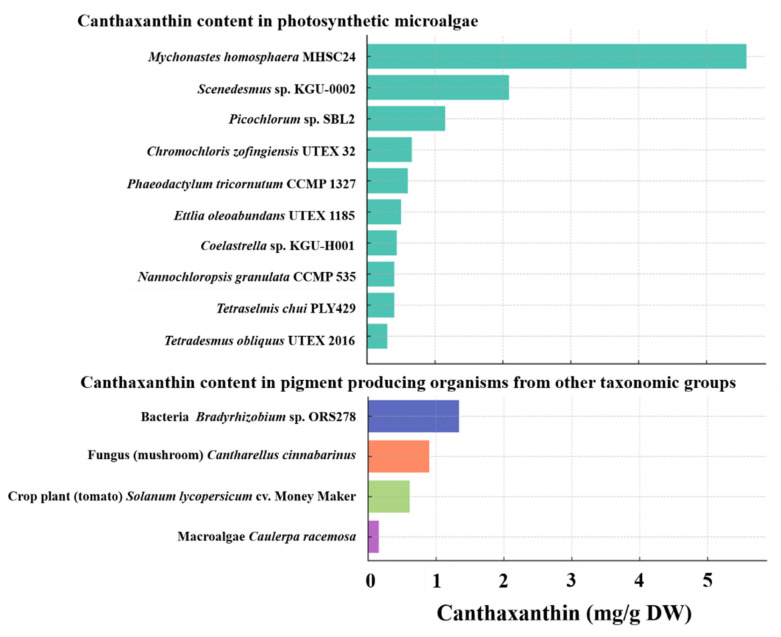
Canthaxanthin content (mg g^−1^ DW) in *M*. *homosphaera* MHSC24 compared with that in selected photosynthetic microalgae (**top**) and pigment-producing organisms from other taxonomic groups (**bottom**), including bacteria, fungi (mushroom), macroalgae, and crops. Representative species include *Scenedesmus* sp. KGU-0002 [68], *Picochlorum* sp. SBL2 [69], *Chromochloris zofingiensis* UTEX 32 [70], *Phaeodactylum tricornutum* CCMP 1327 [71], *Ettlia oleoabundans* UTEX 1185 [71], *Coelastrella* sp. KGU-H001 [68], *Nannochloropsis granulata* CCMP 535 [71], *Tetraselmis chui* PLY429 [71], *Tetradesmus obliquus* UTEX 2016 [71], *Bradyrhizobium* sp. ORS278 [72], *Cantharellus cinnabarinus* [73], *Solanum lycopersicum* cv. Money Maker [74], and *Caulerpa racemosa* [75].

The canthaxanthin content of MHSC24 (5.59 mg g^−1^ DW) exceeded those reported for several green microalgal strains cultivated under standard conditions, including *Scenedesmus* sp. KGU-0002 (2.09), *Picochlorum* sp. SBL2 (1.15), and *Chromochloris zofingiensis* UTEX 32 (0.658). In contrast, many other strains remained below 0.5 mg g^−1^ DW (Figure 10). MHSC24 surpassed pigment-producing organisms from other taxa, such as *Bradyrhizobium* sp. ORS278 (1.34), *Cantharellus cinnabarinus* (0.9), transgenic tomato *Solanumlycopersicum* (0.6115), and the macroalga *Caulerpa racemosa* (0.157). These results highlight its potential as a canthaxanthin producer.

Unlike many organisms where canthaxanthin accumulation requires stress induction or genetic modification [76,77,78,79], MHSC24 achieved high yields under standard cultivation conditions. This indicates that MHSC24 is an efficient biological producer capable of increasing pigment levels without external stimuli. Nevertheless, evidence from other microalgae (e.g., *Chlorella zofingiensis* under salinity or light stress [80] and *Graesiella emersonii* under heterotrophic cultivation [81]) suggests that environmental or trophic modulation could further enhance productivity. This demonstrates the need for targeted optimization studies.

In addition to its biosynthetic strength, canthaxanthin has antioxidant, anti-inflammatory, anticancer, and immunomodulatory properties [7]. The global canthaxanthin market, valued at approximately USD 135 million in 2023, is projected to grow at a Compound Annual Growth Rate (CAGR) > 2.6% by 2032. This growth is driven by the increasing demand for cosmetics, personal care, and animal feed [82]. Its established role in enhancing the coloration of egg yolks, poultry, and aquaculture species (e.g., trout and salmon), together with its recognized health benefits, underpin this growing demand.

In summary, MHSC24 combines superior pigment productivity with cultivation stability, making it a promising candidate for commercial canthaxanthin production. Its potential applications span functional foods, nutraceuticals, cosmetics, and animal feed, underscoring its value as a sustainable platform for natural pigment biomanufacturing amid increasing industrial demand.

### 4.6. Lipid and Carbohydrate Composition and Implications for Functional Applications

The biochemical analysis of *M. homosphaera* MHSC24 under optimal growth conditions revealed relatively low levels of total lipids (9%) and carbohydrates (12%) (Figure 9). Although the total lipid levels were modest, the fatty acid profile (Table 5) revealed a favorable composition enriched in nutritionally and industrially relevant unsaturated fatty acids. The predominant species were oleic acid (C_18:1_ n-9; 17.28%, ω-9 MUFA), α-linolenic acid (C_18:3_ n-3; 15.61%, ω-3 PUFA), and linoleic acid (C_18:2_ n-6; 6.28%, ω-6 PUFA). This composition highlights its potential use in nutraceutical and functional foods [83,84], although the overall lipid yield may constrain immediate large-scale applications in lipid-derived bioindustries.

The monosaccharide profile (Table 6) also indicated low sugar accumulation, with galactose (2.068 mg g^−1^ DW) as the dominant component, followed by sucrose (0.990 mg g^−1^ DW) and glucose (0.749 mg g^−1^ DW). These values are considerably lower than those of carbohydrate-rich taxa, such as *Chlorella salina* and *Dunaliella salina*.

Although glucose is generally reported as the predominant monosaccharide in Chlorophyta microalgae, galactose was the major sugar in MHSC24. This finding is not unprecedented; a previous study identified galactose as a principal monosaccharide in certain Chlorophyta species [85]. Therefore, the relatively high galactose content in MHSC24 may reflect species-specific carbohydrate metabolism and suggests that the sugar composition in microalgae is more diverse than generally theorized.

Despite these limitations, many microalgae enhance lipid and carbohydrate accumulation under stress-inducing conditions such as nitrogen limitation, high irradiance, or shifts in salinity. Therefore, future studies should evaluate cultivation strategies for MHSC24 to improve biochemical productivity and expand its potential utility in industrial biotechnology.

In the present study, *M*. *homosphaera* MHSC24 was identified as a distinct strain through integrated morphological and phylogenetic analyses, marking the first report of an indigenous *M*. *homosphaera* strain in Korea. This discovery expands the known biogeographic range of the species and provides a valuable basis for biodiversity research and biotechnological exploration. MHSC24 exhibited characteristics that highlight its potential as a sustainable source of protein-rich biomass and natural antioxidant carotenoids, indicating promising opportunities in food, feed, and health-related sectors. However, further research is required to optimize cultivation strategies, including alternative modes and stress-induced conditions. This optimization could maximize the biotechnological potential of MHSC24 and position it as a versatile platform for industrial applications.

## Figures and Tables

**Table 1:** Marker genes, amplicon lengths, and GenBank accession numbers (GBAN) of *M. homosphaera* MHSC24.

Species	Strain	Marker Gene	Amplicon Length (bp)	GBAN
*M. homosphaera*	MHSC24	SSU	1619	PV432769
		ITS1-5.8S-ITS2	739	PV432770
		LSU	827	PV432771
		*rbc*L	376	PV430742
		*tuf*A	873	PV430743

Small-subunit ribosomal DNA (SSU), internal transcribed spacer (ITS), large subunit ribosomal DNA (LSU), ribulose-1,5-bisphosphate carboxylase/oxygenase large subunit (*rbc*L), translation elongation factor Tu gene (*tuf*A).

**Table 2:** Morphological and ultrastructural characteristics of *M. homosphaera* strains.

Characteristics	MHSC24	CCAP 211/8e	ND
Strain locality	Korea	Sweden	Israel
Habitat	Brackish water	Freshwater	Freshwater
Cell shape	Spherical to ovoid (young & mature)	Spherical to ellipsoidal	Spherical to ovoid (mature)
Young cell size (μm)	1.43–2.67 (2.10 ± 0.08, spherical) 1.25–2.75(1.93 ± 0.08) × 1.00–2.38(1.61 ± 0.07, ovoid)	ND	1.0–2.5 (spherical) 1.0–2.5 × 1.5–3.0 (ovoid)
Mature cell size (μm)	2.78–4.02 (3.46 ± 0.07, spherical) 2.63–3.88 (3.04 ± 0.07) × 2.38–3.63 (2.76 ± 0.07, ovoid)	1.5–5.7 (spherical)	1.5–4.5 (spherical) 1.5–3.5 × 2.0–4.5 (ovoid)
Chloroplast	Parietal, cup-shaped; no pyrenoid	Parietal, cup-shaped; no pyrenoid	Parietal, cup or mantel shaped; no pyrenoid
Nucleus	Single, lateral	Single, near-central *	Single, lateral
Reproduction	Autosporic, 2–4 (rarely 6) autospores	Autosporic, 2–4 (occasionally 8–16) autospores	Autosporic, 2–8 (<16) autospores
Cell wall	Double-layered; irregular ribs/undulations	Double-layered; irregular network of ribs	Double-layered; irregular network of ribs
Plastoglobules	Present in chloroplast	ND	Present in chloroplast
Lipid globules	Present in cytoplasm	ND	Present in cytoplasm
References	This study	[13]	[24]

ND: information not available; * Not mentioned but observed in the figures.

**Table 3:** Comparison of nuclear rDNA regions (SSU, ITS1–5.8S–ITS2, and LSU) and chloroplast genes (*rbc*L and *tuf*A) of *M. homosphaera* MHSC24 isolated from Janghang Harbor, Korea, with those of other strains.

Marker Gene	Collection Location	Strain Habitat (Isolation Source)	Strain Name	GenBank Accession No.	*M. homosphaera* MHSC24 *
SSU	Czech Republic	Freshwater	CCALA 380	GU799582	0 (0)
	Sweden	Freshwater	CAUP H6501	GU799581	0 (0)
	Israel	Freshwater	ND	AB025423	0 (0)
	Germany	Freshwater	CCAP 205/1	GQ477054	1 (0.1)
ITS1-5.8S-ITS2	Japan	Freshwater	NIES-4546	LC853079	0 (0)
	Japan	Freshwater	NIES-2341	LC853071	2 (0.3)
	Germany	Freshwater	CCAP 205/1	GQ477054	7 (0.9)
	Czech Republic	Freshwater	CCALA 380	GU799582	12 (1.6)
	Sweden	Freshwater	CAUP H6501	GU799581	22 (2.9)
LSU	ND	ND	CAUP H6502	KC145446	2 (0.3)
*rbc*L	ND	ND	CAUP H6502	KC145515	0 (0)
	Japan	Freshwater	NIES-2341	LC853338	0 (0)
	Japan	Freshwater	NIES-4546	LC853346	0 (0)
	South Africa	Dump soils	GA11	MW363988	0 (0)
	South Africa	Dump soils	GA13	MW363990	1 (0.3)
*tuf*A	ND	ND	CAUP H6502	KC145523	3 (0.3)
	South Africa	Dump soils	GA13	MW363978	5 (0.6)

* Numbers indicate the number of base pairs that differed between *M*. *homosphaera* MHSC24 and other strains. Numbers in parentheses indicate dissimilarities (%), including gaps. ND: Information not available.

**Table 4:** Photosynthetic pigment composition of *M. homosphaera* MHSC24 cultivated at optimal salinity (0 PSU).

Pigments	Retention Time	Peak Area	Amount (mg g^−1^ DW) *
Peridinin	16.602	1.06	0.27 ± 0.002
Neoxanthin	23.337	11.68	0.48 ± 0.093
Violaxanthin	24.529	19.18	0.61 ± 0.065
Antheraxanthin	28.148	4.03	0.13 ± 0.058
Zeaxanthin	30.118	52.79	0.95 ± 0.210
Lutein	30.315	224.47	3.64 ± 0.834
Canthaxanthin	31.272	219.32	5.59 ± 0.359
Chlorophyll *b*	36.279	64.39	2.50 ± 0.483
Echinenone	37.896	22.53	0.50 ± 0.033
Chlorophyll *a*	39.039	96.52	11.42 ± 2.663
Pheophytin *a*	41.719	1.17	1.33 ± 0.207
β-carotene	42.422	31.33	0.49 ± 0.094

* Values represent the mean ± SD of three independent experiments. Amounts are expressed as mg g^−1^ dry weight (DW).

**Table 5:** Fatty acid composition of *M. homosphaera* MHSC24 cultivated at optimal salinity (0 PSU).

Component	Content (mg g^−1^ DW)	Content (%)	Note
Palmitic acid (C_16:0_)	11.95 ± 0.36	39.72 ± 0.6	SFA (major)
Stearic acid (C_18:0_)	0.65 ± 0.02	2.17 ± 0.03	
Oleic acid (C_18:1_ n-9)	5.2 ± 0.16	17.28 ± 0.26	ω-9 MUFA (major)
Linoleic acid (C_18:2_ n-6)	1.89 ± 0.06	6.28 ± 0.09	ω-6 PUFA (major)
α-linolenic acid (C_18:3_ n-3)	4.7 ± 0.14	15.61 ± 0.23	ω-3 PUFA (major)
Eicosapentaenoic acid (C_20:5_ n-3)	0.27 ± 0.01	0.88 ± 0.01	
Lignoceric acid (C_24:0_)	3.33 ± 0.1	11.07 ± 0.17	
Nervonic acid (C_24:1_)	2.1 ± 0.06	6.98 ± 0.11	
Total saturated fatty acids	15.93	52.96	
Total monounsaturated fatty acids	7.3	24.26	
Total polyunsaturated fatty acids	6.8	22.77	

Data are expressed as mean contents (mg g^−1^ DW) and percentages of total fatty acids. SFA, saturated fatty acid; MUFA, monounsaturated fatty acid; PUFA, polyunsaturated fatty acid.

**Table 6:** Comparative monosaccharide composition (mg g^−1^ DW) of *M. homosphaera* MHSC24 and selected microalgae species.

Species	Strain	Monosaccharides (mg g^−1^ DW)								Reference
		Arabinose	Fructose	Galactose	Glucose	Lactose	Mannitol	Sorbitol	Sucrose	
*Mychonastes homosphaera*	MHSC24	-	0.617 ± 0.031	2.068 ± 0.103	0.749 ± 0.037	-	0.457 ± 0.023	-	0.990 ± 0.05	This study
*Chlorella salina*	MM0063	27.8	19.0	75.1	124.1	-	3.5	5.6	-	[47]
*Dunaliella salina*	DSTA20	-	13.2	15.7	195.5	-	-	-	7.13	[36]
*D. tertiolecta*	CS-175	0.65	-	1.1	85.3	-	-	-	-	[48]
*Picochlorum atomus*	CS-183	0.16	-	10.6	55.2	-	-	-	-	[48]

Values are expressed as mg g^−1^ DW. “-” indicates data that are not available or below detection limits.

## Data Availability

The original data presented in this study are openly available from the National Marine Biodiversity Institute of Korea and the Korean Collection for Type Cultures under the accession numbers MABIK FL00031124 and KCTC 16415BP, respectively.

## Supplementary Materials

The following supporting information can be downloaded at: https://www.mdpi.com/article/10.3390/microorganisms13102322/s1, Figure S1: Maximum likelihood (ML) and Bayesian inference (BI) phylogenetic tree based on ITS1–5.8S–ITS2 rDNA sequences.; Figure S2: Maximum likelihood (ML) and Bayesian inference (BI) phylogenetic tree based on *rbc*L sequences.
