# Supplementary material for: Mychonastes homosphaera MHSC24 Isolated from Brackish Waters of Korea: Taxonomic, Physiological, and Biochemical Characterization
Source: Microorganisms. 2025 Oct 7;13(10):2322. doi: 10.3390/microorganisms13102322 (PMC12565954; doi:10.3390/microorganisms13102322)
Supplement: Supplementary file 1 [file microorganisms-13-02322-s001.zip › microorganisms-3897154-supplementary.pdf]

microorganisms

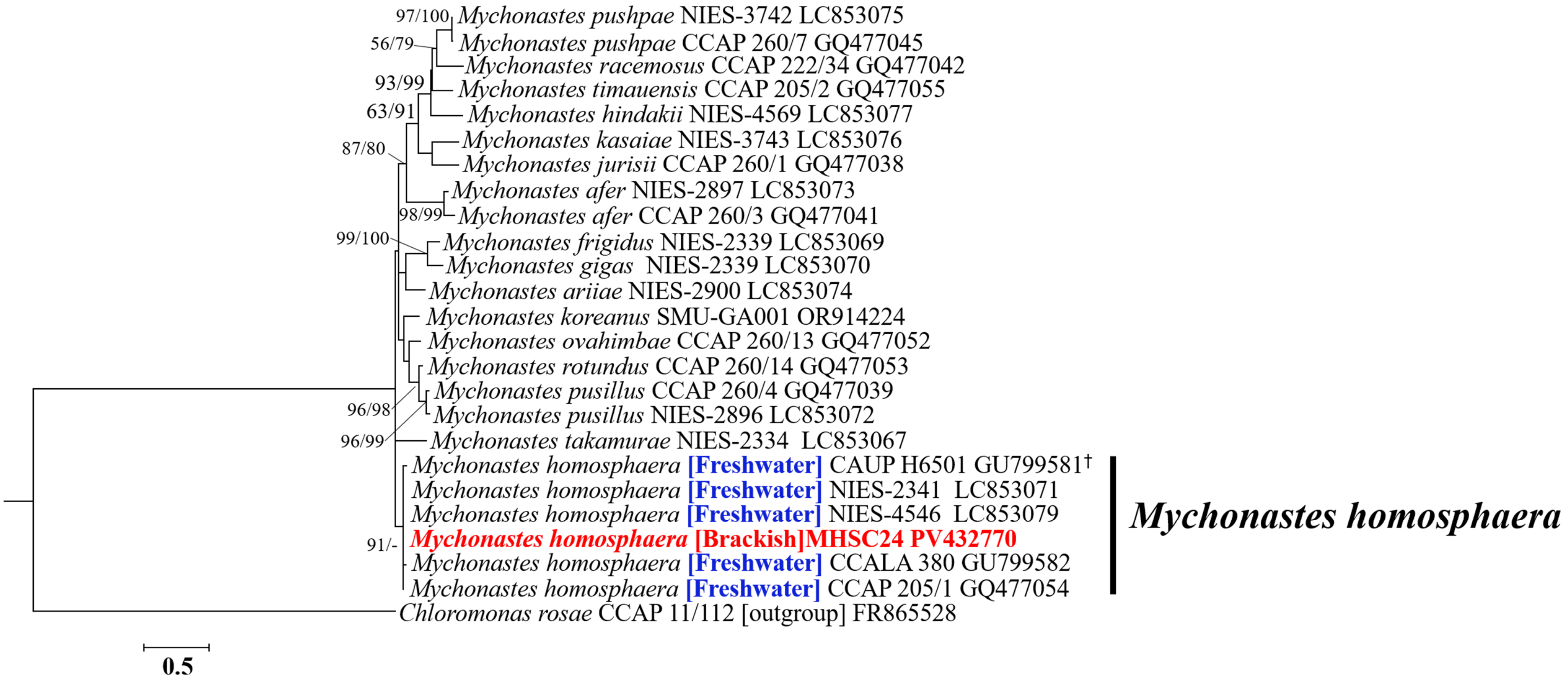

Figure S1. Phylogenetic tree inferred by ML and BI based on ITS1–5.8S–ITS2 rDNA sequences. The numbers at each node indicate ML bootstrap values (left) and BI posterior probabilities (right). Bootstrap values < 50% are not shown; posterior probabilities are indicated for major nodes, with an asterisk (*) representing full support (1.00). The strain analyzed in this study (*M. homosphaera* MHSC24) is highlighted in red. Blue labels indicate freshwater strains. A dagger symbol (†) denotes the authentic strain. The scale bar represents the number of nucleotide substitutions per site.

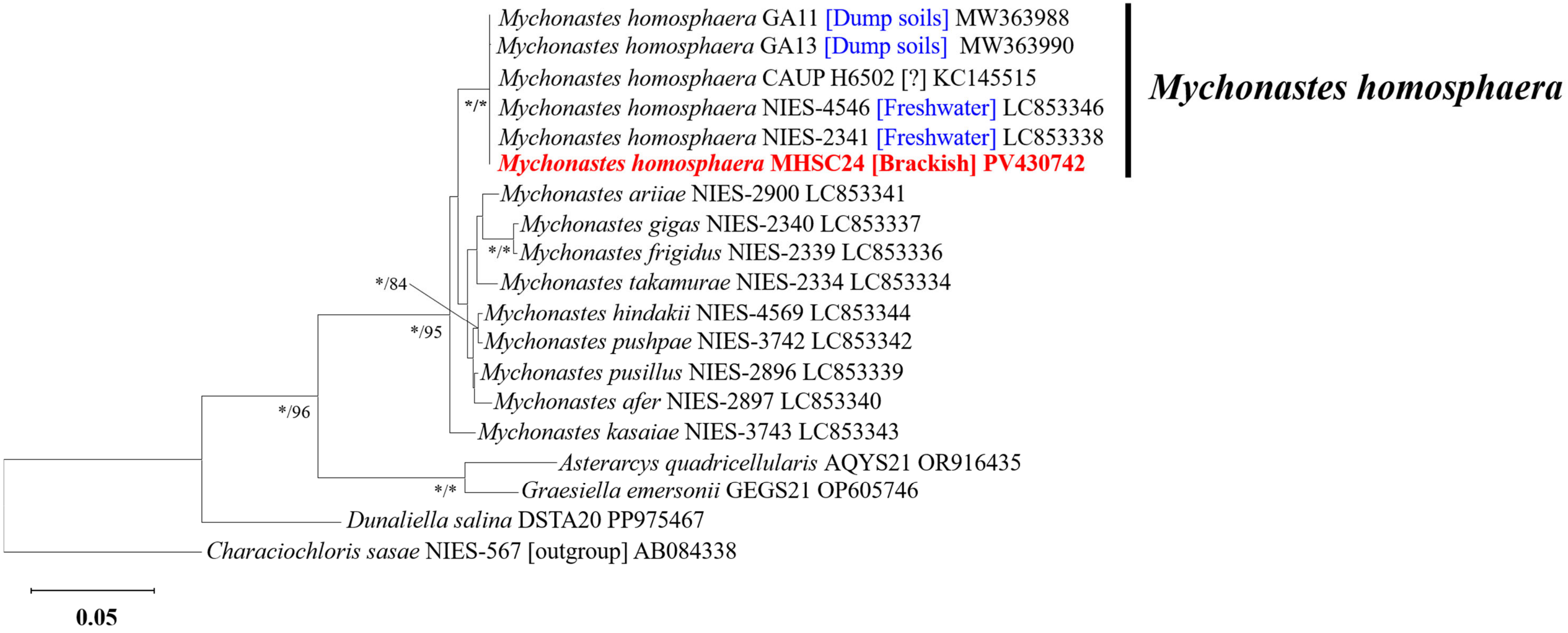

Figure S2. Phylogenetic tree inferred by ML and BI based on *rbc*L sequences. The numbers at each node represent ML bootstrap values (left) and BI posterior probabilities (right). Bootstrap values < 50% are not shown; posterior probabilities are shown for major nodes, with an asterisk (*) indicating full support (1.00). The strain analyzed in this study (*M. homosphaera* MHSC24) is highlighted in red. Blue labels indicate the freshwater strains. Question marks (?) denote strains whose habitat is unknown. The scale bar represents the number of nucleotide substitutions per site.

Disclaimer/Publisher's Note: The statements, opinions and data contained in all publications are solely those of the individual author(s) and contributor(s) and not of MDPI and/or the editor(s). MDPI and/or the editor(s) disclaim responsibility for any injury to people or property resulting from any ideas, methods, instructions or products referred to in the content.
